# Reducing the Glucose Level in Pre-treatment Solution Improves Post-thaw Boar Sperm Quality

**DOI:** 10.3389/fvets.2022.856536

**Published:** 2022-03-30

**Authors:** Zhendong Zhu, Weijing Zhang, Rongnan Li, Wenxian Zeng

**Affiliations:** ^1^College of Animal Science and Technology, Qingdao Agricultural University, Qingdao, China; ^2^College of Animal Science and Technology, Northwest A&F University, Yangling, China

**Keywords:** boar sperm, cryopreservation, glycolysis, quality, metabolism

## Abstract

Frozen–thawed boar sperm was not widely used in pig artificial insemination as the sperm quality was damaged by biochemical and physical modifications during the cryopreservation process. The aim of this study was to investigate whether reduction of the glucose level in diluted medium could protect the post-thaw boar sperm or not. Boar sperm was diluted with the pre-treatment medium with different doses of glucose (153, 122.4, 91.8, 61.2, 30.6, and 0 mM) during the cooling process. The sperm motility patterns and glycolysis were evaluated during the cooling process. Meanwhile, the post-thaw sperm quality, ATP level, mitochondrial function as well as apoptosis were also measured. It was observed that 153 mM glucose treatment showed the highest glycolysis in boar sperm as the activities of hexokinase, fructose-bisphosphate aldolase A, and lactate dehydrogenase are the highest as well as the lactate level. Reduction of the glucose level from 153 to 30.6 mM suppressed sperm glycolysis. In addition, treatment with 153 mM glucose made the sperm demonstrate a circle-like movement along with a high value of curvilinear velocity and amplitude of the lateral head, while decreasing the glucose level reduced those patterns in the cooling process. Moreover, reduction of the glucose level also significantly increased the post-thaw sperm's total motility, progressive motility, straight-linear velocity, membrane integrity, and acrosome integrity. The treatment with 30.6 mM glucose showed the highest value among the treatments. Furthermore, the post-thaw sperm's succinate dehydrogenase activity, malate dehydrogenase activity, mitochondrial membrane potential as well as ATP level were increased by reducing the glucose level from 153 to 30.6 mM. Interestingly, the treatment with 30.6 mM glucose showed the lowest apoptosis of post-thaw sperm among the treatments. Those observations suggest that reduction of the glucose level in diluted medium increased the post-thaw boar sperm quality *via* decreasing the glycolytic metabolism. These findings provide novel insights that reduction of boar sperm activity *via* decreasing sperm glycolysis during the cooling process helps to improve the post-thaw sperm quality during cryopreservation.

## Introduction

Sperm cryopreservation is one of the most essential assisted reproductive techniques in the livestock industry. During the cooling, freezing, and thawing processes, the sperm suffers from lots of dramatic changes in its physical and chemical surroundings (1, 2) that cause phase transitions of the membrane lipids (3) and impair cellular metabolism homeostasis. Previous studies found that cryo-damages affect post-thaw boar sperm fertility (4, 5). In order to increase the fertilization rates of artificial insemination (AI) with the post-thaw boar sperm, several research groups modify the cryopreservation extender, cooling temperature program or develop a sperm infusion method (2, 6). Though AI with post-thaw boar sperm could achieve a high conception rate of about 70% recently (7), the frozen–thawed boar sperm has not been widely accepted by commercial pork production. Only <1% of artificial inseminations are done with boar frozen–thawed sperm (6) because the birth rate and litter size in artificial insemination with post-thaw semen could not exceed 75–80% and 9–11.5 live births (8, 9) when compared to the utilization of liquid-stored semen at 17°C which is 20–30% more efficient in birth rate and results in 2 to 3 more young born per litter (8). In addition, the lifespan of post-thaw boar sperm in the female reproductive tracts is much shorter than that of fresh semen (10), which is also a major reason for the limitation of the post-thaw boar sperm application. Hence, novel strategies to maintain post-thaw boar sperm motility and function are important for the application of AI with frozen–thawed sperm in the swine industry.

In mammalian sperm, glycolysis and mitochondrial oxidative phosphorylation (OXPHOS) are the two major metabolic pathways for generating ATP (11, 12). Glycolysis occurs along the entire length of the principal piece of the flagellum as the glycolysis-related enzymes were distributed in those regions (12–14). Meanwhile, the OXPHOS occurs on the sperm mid-piece where the mitochondrion was located (15, 16). Sperm metabolism could be altered by its surrounding environment (14). A high-glucose medium stimulates sperm glycolysis and activates boar sperm motility patterns with circle-like tracts (17). Interestingly, the glucose level in boar fresh seminal plasma was very low (18). In addition, during cryopreservation, the sperm was cooled before the freezing process to lower down the sperm activity due to the fact that activated sperm is not a good condition for freezing (19). In somatic cell or organ cryopreservation, decreasing the cellular metabolic activity is beneficial to increase the cell and organ quality after recovering from cryopreservation (20, 21). However, the Modena solution, which contained 153 mM glucose, is usually regarded as the pre-treatment medium before the boar semen was cooled to 15°C during the cryopreservation process (19). Furthermore, in our previous study, we found that the reduction of glucose level in the incubation medium led to a decrease in boar sperm glycolysis metabolism (17). Therefore, we hypothesize that decreasing the glucose level in the pre-treatment medium might enhance the post-thaw boar sperm quality *via* reducing sperm glycolysis to protect the sperm during cryopreservation.

## Materials and Methods

### Chemicals and Extenders

All chemicals and reagents were purchased from Sigma unless specified otherwise.

The Modena solution was composed of 153 mM D-glucose, 26.7 mM trisodium citrate, 11.9 mM sodium hydrogen carbonate, 15.1 mM citric acid, 6.3 mM EDTA-2Na, 46.6 mM Tris, 1,000 IU/ml Penicillin G Sodium Salt, 100 μg/ml polymyxin B, and 1 mg/ml streptomycin. According to our previous study (17), lactose was used instead of glucose to make different doses of glucose pre-treatment extenders (153, 122.4, 91.8, 61.2, 30.6, and 0 mM). As described by previous studies (19, 22), the modified Niwa and Sasaki freezing extender (mNSF1) was prepared with 80% (v/v) of 0.31 M lactose monohydrate, 20% (v/v) of egg yolk, 1,000 IU/ml Penicillin G Sodium Salt, and 100 μg/ml polymyxin B. The second dilution (mNSF2) was made with mNSF1 containing 1.5% (v/v) Orvus Es Paste (Miyazaki Chemical Sales, Ltd., Tokyo, Japan) and 2% (v/v) glycerol. Modena solution was also used as thawing solution.

### Semen Collection and Processing

Five mature and fertile Duroc boars (aged 2 years) were used in the present study. All animal treatments and experimental procedures were approved by the Qingdao Agricultural University Institutional Animal Care and Use Committee (QAU-1121010). A sperm-rich fraction was collected weekly from each boar using the gloved-hand technique and filtered through double gauze. Only the ejaculates containing sperm with more than 90% motility and 85% normal morphology were used in this study. The ejaculated semen was pooled to avoid individual differences.

According to our previous study (22), fresh semen was divided into 6 parts and directly diluted with the pre-treatment solution with different doses of glucose level (v:v = 1:1) for 2 h at 15°C. After that, the samples were centrifuged for 10 min at 700 × *g* to remove the pre-treatment solutions. The sperm pellets were resuspended with mNSF1 at a concentration of 2.0 × 10^9^ sperm/ml and cooled from 15 to 5°C with 1.5 h. Subsequently, the sperm sample was mixed with mNSF2 (v/v = 1:1) and packed into 0.5-ml plastic straws. The straws were placed in liquid nitrogen vapor for 10 min, plunged into it for storage. The straws were transferred to water at 60°C for 8 s to thaw the frozen sperm and diluted with 4.5 ml of thawing solution per straw.

### Evaluation of Sperm Motility, Membrane Integrity, and Acrosome Integrity

Sperm motility was measured using a computer-assisted sperm motility analysis (CASA) system (HT CASA-Ceros II; Hamilton Thome, MA, USA). Briefly, a 5-μl aliquot of semen was placed on the analyzer's Makler chamber and maintained at 37°C during the analysis. Three fields were selected for computer-assisted analysis; more than 500 sperms were evaluated (17).

Sperm membrane integrity and acrosome integrity were, respectively, evaluated using LIVE/DEAD Sperm Viability Kit (L7011; Thermo Fisher Scientific) and fluorescein isothiocyanate (FITC)–peanut agglutinin according to our previous study (23). The stained sperm was monitored and photographed by an epifluorescence microscope (Nikon 80i; Tokyo, Japan) with a set of filters (×200).

### Evaluation of Sperm Mitochondrial Membrane Potential

JC-1 Mitochondrial Membrane Potential Detection Kit (Beyotime Institute of Biotechnology, China) was used to analyze the changes of sperm mitochondrial activity (ΔΨm) (24). The monomer and aggregates of the two types of JC-1 in stained mitochondrial plasma emit green fluorescence in low ΔΨm and emit red fluorescence in high ΔΨm, respectively. Briefly, sperm samples (2 × 10^6^/ml) were stained with 1 × JC-1 at 37°C for 30 min. The fluorescence intensity of both mitochondrial JC-1 monomers and aggregates was detected with a monochromator microplate reader (Safire II, Tecan, Switzerland). The Δψm of sperm in each treatment group was calculated as the fluorescence ratio of red (aggregates) to green (monomer). The analyses were performed in triplicate (*n* = 3).

### Analysis of Hexokinase Activity

According to a previous study (25), a 2-deoxyglucose Uptake Measurement Kit was used to measure the sperm hexokinase activity (Cosmo Bio, Japan). The sperm sample pellets were mixed with the reaction mix in a 96-well microplate. The absorbance was measured with a multimode plate reader at 420 nm. The 2DG6P level was calculated using a standard curve.

### Measure of Fructose-Bisphosphate Aldolase a Activity

Sperm ALDOA activity was measured with an aldolase activity colorimetric assay kit (BioVision, K665-100) (25). The sperm sample was homogenized with aldolase assay buffer and centrifuged to collect supernatant. Then, the supernatants were used to analyze ALDOA activity in a 96-well plate. The absorbance was measured with a multimode plate reader at 450 nm according to the manufacturer's instructions.

### Measure of LDH Activity

Lactate dehydrogenase assay kit (Nanjing Jiancheng Bioengineering Institute, China) was used to measure the lactate dehydrogenase (LDH) activity. According to the manufacturer's instructions, sperm sample pellets were lysed ultrasonically (20 kHz, 750 W, operating at 40% power, 5 cycles of 3 s on and 5 s off) and centrifuged at 2,000 × *g* for 10 min at 4°C. The supernatants were added to a 96-well plate for the analysis of LDH activity with a microplate reader at 450 nm (26). The analyses were performed in triplicate (*n* = 3).

### Detection of Lactate Level

Sperm lactate content was measured with a lactate detection kit (Nanjing Jiancheng, China) according to the manufacturer's instruction. Briefly, the level of lactate was measured by detecting the NADH formed consequently to lactate oxidation by LDH with a microplate reader at 340 nm (27).

### Measure of MDH and SDH Activities

According to our previous study, Malate Dehydrogenase Assay Kit and Succinate Dehydrogenase Assay kit (Nanjing Jiancheng Bioengineering Institute, China) were used to measure the activities of malate dehydrogenase (MDH) and succinate dehydrogenase (SDH), respectively. The sperm sample pellets were lysed ultrasonically (20 kHz, 750 W, operating at 40% power, 5 cycles of 3 s on and 5 s off) and centrifuged at 2,000 × *g* for 10 min at 4°C to collect the supernatant. Then, the supernatant was added to the 96-well plate for evaluation of MDH and SDH activities with a microplate reader at 340 and 600 nm, respectively. The analyses were performed in triplicate (*n* = 3).

### Measure of Sperm ATP Level

ATP Assay Kit (Beyotime Institute of Biotechnology) was used to measure the post-thaw sperm ATP level according to our previous study (26). After lysis and centrifugation, 50 μl of the sperm supernatant was mixed with 100 μl luciferin/luciferase reagent in 96-well plates. An Ascent Luminoskan luminometer (Thermo Scientific, Palm Beach, FL, United States) was used to read the luminescence at integration × 1,000 ms. The analyses were performed in triplicate (*n* = 3).

### Annexin V-FITC/PI Assay

Annexin V-FITC/PI apoptosis detection kit (Sigma-Aldrich, St. Louis, MO, USA) was used to assess sperm apoptosis according to the manufacturer's instructions but with slight modifications. The post-thaw sperm was centrifuged and washed thrice with phosphate-buffered saline at 400 × *g* for 5 min. The sperm was resuspended with 1× Annexin V binding buffer at a concentration of 1 × 10^6^ sperm/ml. A total of 5 μl Annexin V-FITC (AN) and 3 μl PI was then added to each aliquot of 100-μl sample. The tubes were mixed gently and incubated at room temperature for 10 min in dark. Different labeling patterns of stained sperm were observed and counted with a fluorescence microscope (80i; Nikon) at 400× magnification.

### Statistical Analysis

Data from three replicates were compared using either Student's *t*-test or one-way analysis of variance followed by Tukey's *post-hoc* test (Statview; Abacus Concepts, Inc., Berkeley, CA). All the values are presented as mean ± standard deviation (SD). Treatments were considered statistically different from one another at *p* < 0.05.

## Results

### High Glucose Level in the Pre-treatment Medium Activated the Glycolytic Metabolism and Decreased the Membrane Integrity and Acrosome Integrity in Boar Sperm During the Cooling Process

**Figure 1:**
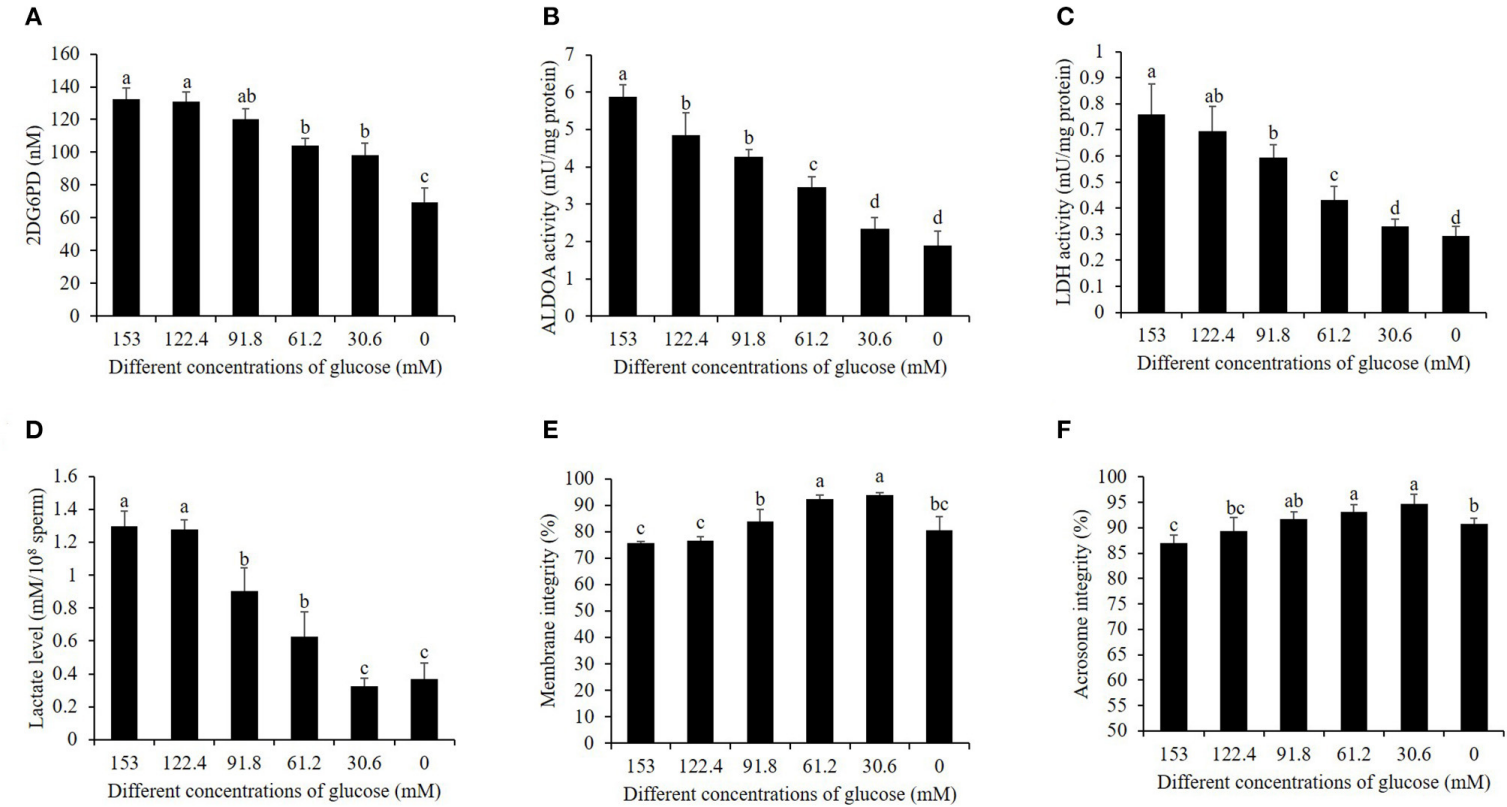
Effects of different glucose levels in the pre-treatment solution on boar sperm hexokinase activity **(A)**, ALDOA activity **(B)**, LDH activity **(C)**, LDH level **(D)**, membrane integrity **(E)**, and acrosome integrity **(F)** during the cooling process. Values are specified as mean ± standard deviation. Columns with different lowercase letters differ significantly (*p* < 0.05). ALDOA, aldolase A; LDH, lactate dehydrogenase.

As shown in Figures 1A–C, during the cooling process, the activities of hexokinase, ALDOH, and LDH in 153 mM glucose medium pre-treatment (high glucose) showed the highest value among the treatments. It was observed that the reduction of glucose level in the pre-treatment medium significantly decreased the hexokinase, ALDOH, and LDH activities (Figures 1A–C). In addition, decreasing the glucose level also reduced the value of the lactate level, considering that lactate is one of the products in the sperm's glycolytic pathway (Figure 1D). The values of sperm membrane integrity and acrosome integrity were also improved by a reduction of glucose level during the cooling process (Figures 1E,F).

### High Glucose Level in the Pre-treatment Medium Increased the Ratio of Sperms With Circle-Like Track Patterns and Decreased the Sperm Membrane Integrity and Acrosome Integrity During the Cooling Process

**Figure 2:**
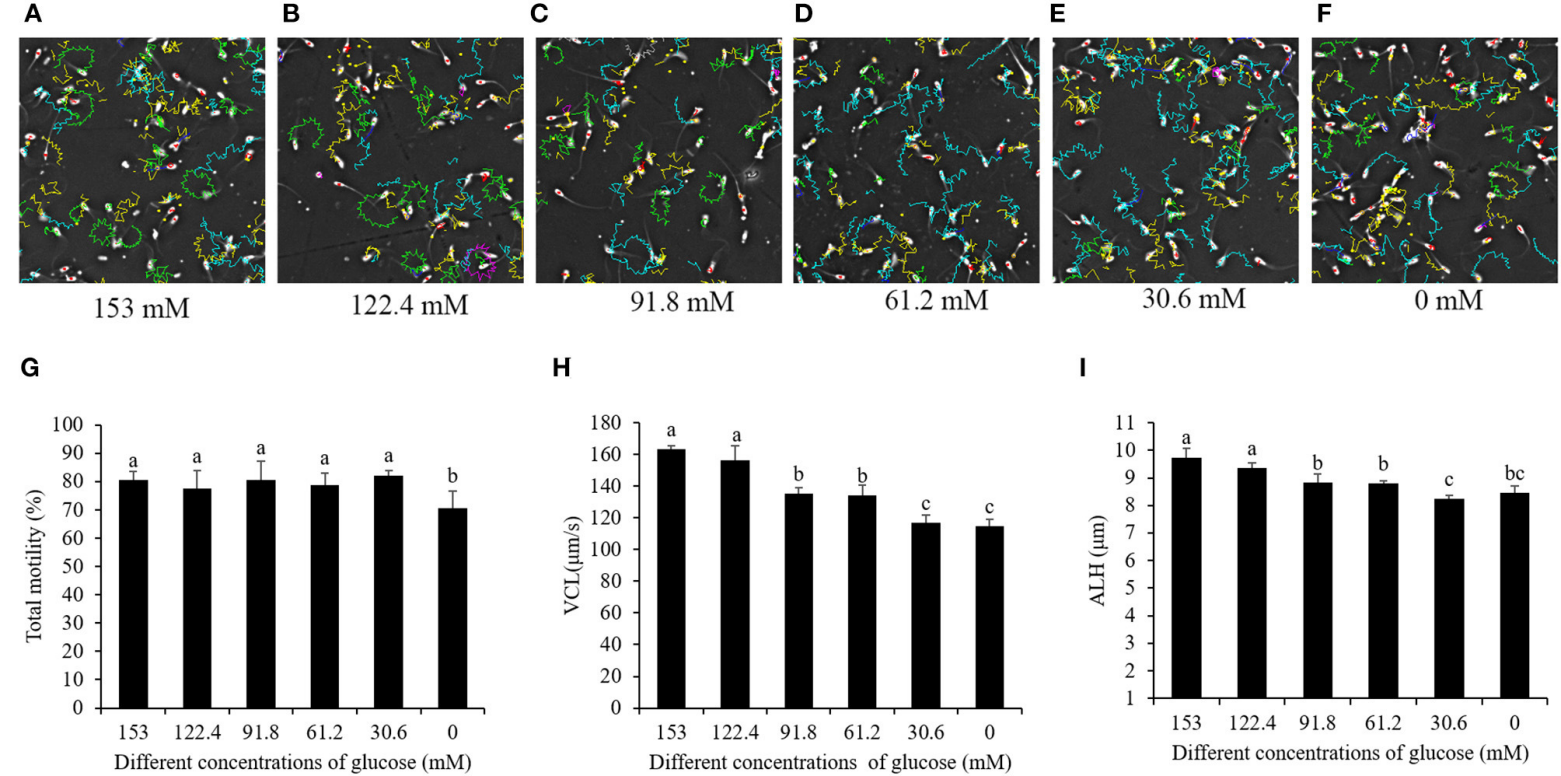
Computer-assisted sperm motility analysis-derived changes in the sperm motility track by reducing the glucose level in the pretreatment solution from 153 to 0 mM **(A–F)**. Effects of glucose level in the pre-treatment solution on sperm total motility **(G)**, VCL **(H)**, and ALH **(I)** during the cooling process. Values are specified as mean ± standard deviation. Columns with different lowercase letters differ significantly (*p* < 0.05). VCL, curvilinear velocity; ALH, amplitude of lateral head displacement.

Interestingly, when the sperm motility patterns were analyzed by CASA, the sperm motility tracks revealed that the high-glucose pre-treatment made the sperm move with circle-like tracks. Meanwhile, the reduction of the glucose level decreased the ratio of sperms with circle-like movement, and the movement changed to a linear-like track pattern during the cooling process (Figures 2A–F). The sperm's total motility was not changed by 153- to 30.6-mM glucose treatments, but treatment with 0 mM glucose significantly decreased it (Figure 2G). Besides this, the patterns of sperm VCL and ALH were also significantly decreased in the treatments where there was a reduction of the glucose level from 153 to 0 mM (Figures 2H,I).

### Reduction of the Glucose Level in Pre-treatment Medium Increased Post-thaw Sperm Motility, Membrane Integrity, and Acrosome Integrity

**Figure 3:**
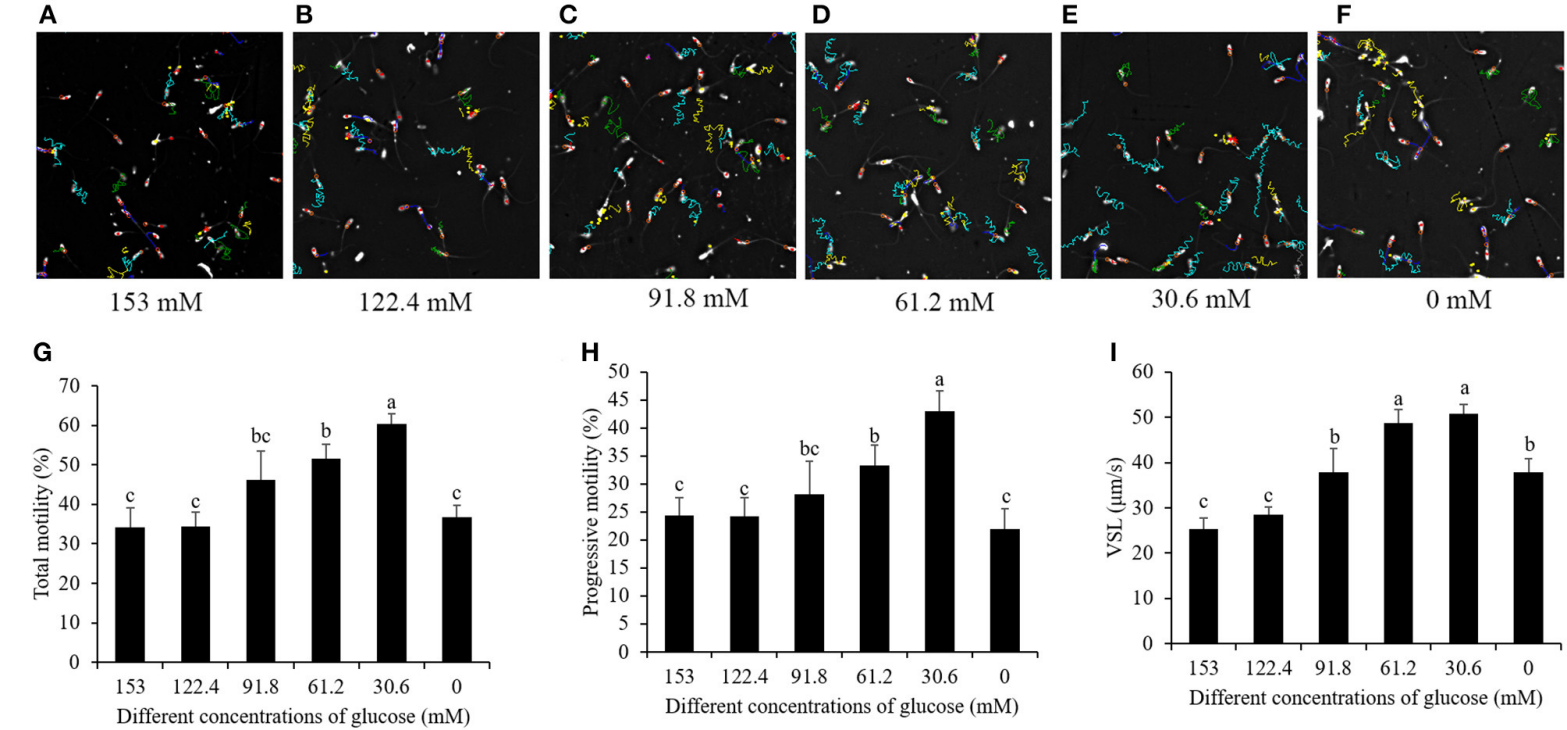
Computer-assisted sperm motility analysis-derived changes of post-thaw sperm motility track by reducing the glucose level in the pretreatment solution from 153 to 0 mM **(A–F)**. Effects of glucose level in the pretreatment solution on post-thaw sperm total motility **(G)**, progressive motility **(H)**, and VSL **(I)**. Values are specified as mean ± standard deviation. Columns with different lowercase letters differ significantly. VSL, straight line velocity.

When the sperm was thawing, from the sperm motility tracks generated from the CASA it was observed that the post-thaw sperm motility patterns were significantly increased by a reduction of the glucose level (Figures 3A–F). The values of sperm total motility, progressive motility, and straight line velocity (VSL) in the treatments with 91.8, 61.2, and 30.6 mM glucose were significantly higher than those in the treatment with 153 mM, and the treatment with 30.6 mM showed the highest value among the treatments (Figures 3G–I). However, the glucose pre-treatment with 0 mM did not increase those motility patterns compared to the high-glucose-medium treatment (Figures 3G–I).

**Figure 4:**
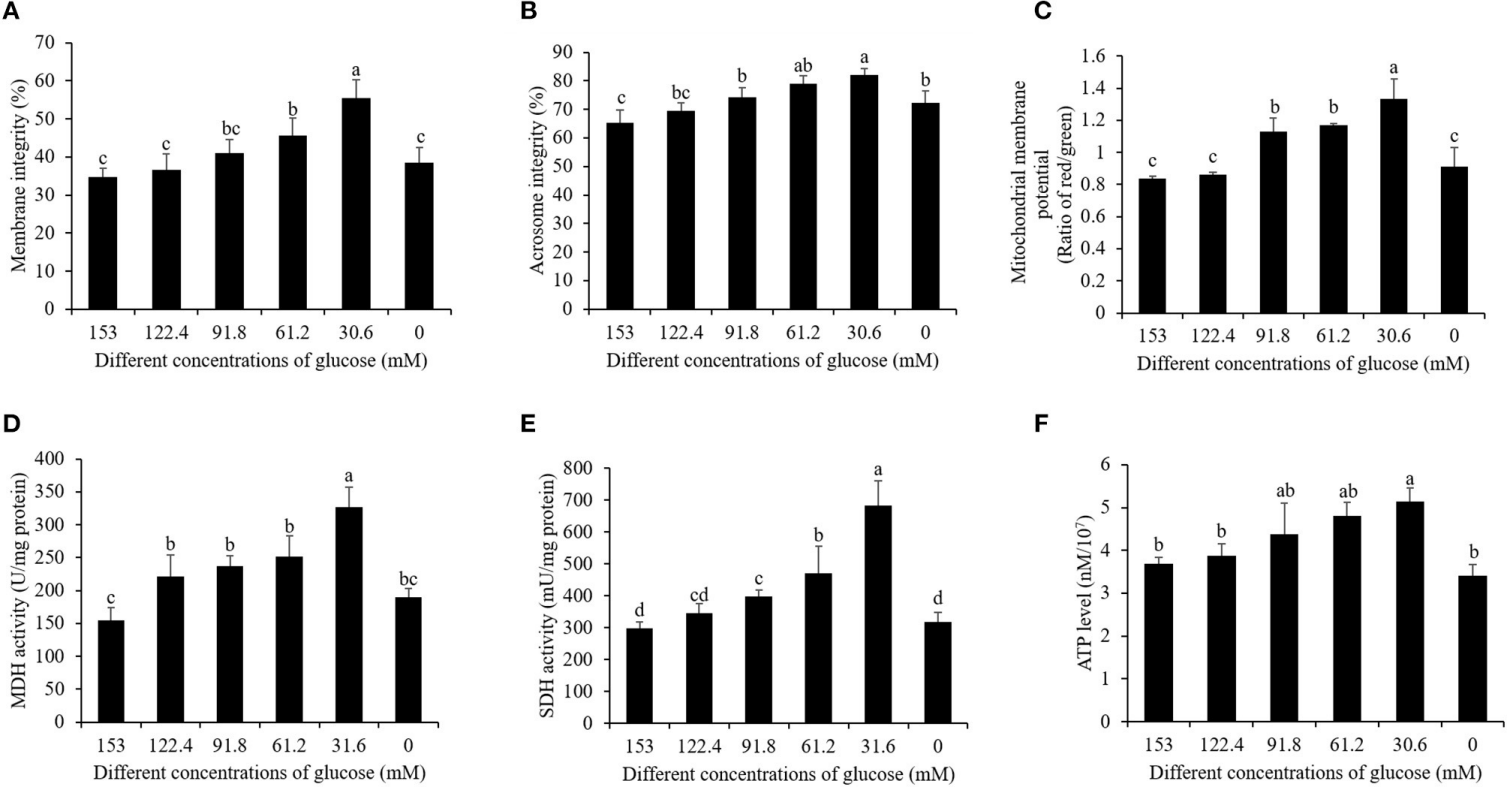
Effects of different glucose levels in the pretreatment solution on boar post-thaw sperm membrane integrity **(A)**, acrosome integrity **(B)**, mitochondrial membrane potential **(C)**, MDH level **(D)**, SDH level **(E)**, and ATP level **(F)** after thawing. Values are specified as mean ± standard deviation. Columns with different lowercase letters differ significantly (*p* < 0.05). MDH, malate dehydrogenase; SDH, succinate dehydrogenase.

In terms of post-thaw sperm membrane integrity and acrosome integrity, the reduction of the glucose level from 153 to 30.6 mM significantly increased them, while the treatment with 0 mM glucose did not increase the membrane integrity (Figures 4A,B). Moreover, the treatment with 30.6 mM glucose presented the best values of post-thaw sperm membrane integrity and acrosome integrity (Figures 4A,B).

### Reduction of the Glucose Level in Pre-treatment Medium Increased Post-thaw Sperm Mitochondrial Function for ATP Generation

**Figure 5:**
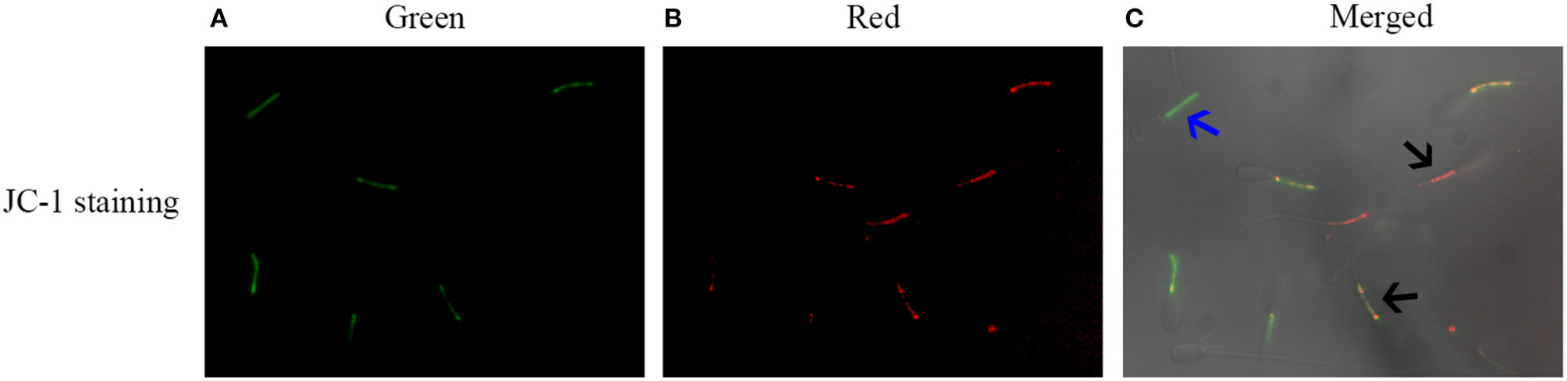
Images of sperm stained with JC-1 probe **(A–C)**. The monomer emits green fluorescence **(A)**, while the aggregates emit red fluorescence **(C)**. The blue arrow indicated the sperm with low mitochondrial membrane potentials, while the black arrow indicated the sperm with high mitochondrial membrane potentials.

Post-thaw sperm mitochondrial membrane potential was measured with JC-1 staining kit. As shown in Figure 5, it was observed that there are two kinds of sperm after staining with the JC-1 probe. The blue arrow showed that the post-thaw sperm emits green fluorescence with low mitochondrial membrane potential, while the black arrow showed that the post-thaw sperm emits red or orange fluorescence with high mitochondrial membrane potential. Reduction of the glucose level during the cooling process significantly increased the post-thaw sperm mitochondrial membrane potential (Figure 4C). Interestingly, the reduction of glucose level during the cooling process also increased the post-thaw sperm activities of MDH and SDH enzymes in which the two are key enzymes of TCA cycle for ATP generation, and the treatment with 30.6 mM glucose showed the best effects (Figures 4D,E). Moreover, the ATP level of the post-thaw sperm was also observed to increase by reduction of the glucose level during the cooling process (Figure 4F).

### Reduction of the Glucose Level in Pre-treatment Medium Decreased Post-thaw Sperm With Apoptosis

**Figure 6:**
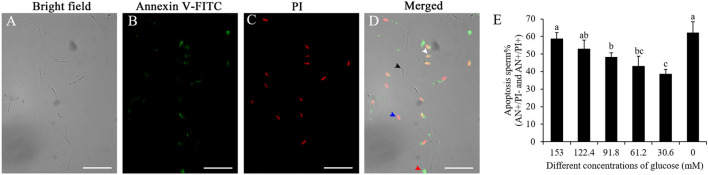
Photos of the boar post-thaw sperm stained with the Annexin V-FITC/PI assay kit **(A–D)**: live sperm (AN-/PI-; black arrow), early apoptotic sperm (AN+/PI–; red arrow), late apoptotic sperm (AN+/PI+; white arrow), and non-viable necrotic sperm (AN–/PI+; blue arrow). Effects of different concentrations of glucose on boar post-thaw sperm apoptosis **(E)**. Values are specified as mean ± standard deviation. Columns with different lowercase letters differ significantly (*p* < 0 05).

As shown in Figures 6A–D, after the sperm samples were stained by the Annexin V-FITC/PI assay kit, four subpopulations of post-thaw sperm were observed: the black arrow indicated the live sperm (AN–/PI–), the red arrow indicated the early apoptotic sperm (AN+/PI–), the white arrow indicated the late apoptotic sperm (AN+/PI+), and the blue arrow indicated the nonviable necrotic sperm (AN–/PI+). Reduction of the glucose level in pre-treatment medium significantly decreased the post-thaw sperm with apoptosis (AN+/PI– and AN+/PI+), and the treatment with 30.6 mM glucose showed the lowest percentage of post-thaw sperm with apoptosis (Figure 6E).

## Discussion

Sperms are special cells with high differentiation that play key roles in paternal DNA delivery and oocyte activation during the fertilization process (28). Boar sperm is deposited in the pig uterus during natural mating or conventional artificial insemination (2, 29). The sperm is physically transported from the site of deposition to the fertilization site, which is located in the oviduct (30). When the sperm transport is facilitated by uterine contractions, the sperm must be with good motility to traverse the uterotubal junction prior to oviduct binding and to locate the egg following ovulation, suggesting that sperm motility is a key factor for improving the chances of fertilization (17, 30). In the present study, we found that decreasing the glucose level in the pre-treatment significantly increased the post-thaw sperm motility, and this might contribute to the improvement of fertilization using frozen boar sperm in artificial inseminations.

Sperm motility depends on ATP production by both the glycolysis in the cytoplasm and oxidative phosphorylation in the mitochondria (11, 14, 17). It is well-known that the sperm contains three distinct regions, namely, head, mid-piece, and tail. Most enzymes involved in glycolysis were located in the sperm head and tail, while those enzymes related with the oxidative phosphorylation pathway were located in the mid-piece where the mitochondria are located (13, 31). In somatic cells, ATP generation occurs by metabolic pathways, depending on oxygen availability and the composition of metabolic substrates in their environment (32, 33). In our previous study (17), during the boar sperm incubation process, ATP generation from the glycolysis pathway decreased when the glucose level in the incubation medium was reduced, which suggests that boar sperm can change the metabolic pathway. Moreover, when HepG2 cells were treated with a medium containing a high level of glucose, the glycolysis pathway was highly activated, and if the glucose level in the medium was reduced, the glycolysis pathway for ATP generation would decrease (34). In the present study, it was observed that, during the cooling process before cryopreservation, the values of the activities of hexokinase, ALDOA, and LDH as well as the lactate level in the high-glucose treatment were significantly higher than those in the low-glucose treatment, suggesting that the high-glucose pre-treatment medium enhanced boar sperm glycolysis, and the reduction of glucose level helped to inactive boar sperm glycolysis during the cooling process. Those results were supported by previous studies which showed that sperm glycolysis metabolism was enhanced when they arrived at the oviduct as the oviduct fluid contained a high glucose level to induce zigzag motility for the sperm to penetrate the oocyte (35). Our previous study revealed that boar sperm energy metabolism could be changed and enhanced depending on the metabolic substrates in their environment. Moreover, the observation on the boar sperm moving with circle-like tracks in high-glucose pre-treatment medium in the present study was also consistent with high-glycolysis-induced boar sperm with high VCL and ALH motility patterns (17). Furthermore, the boar fresh semen contained very low glucose (18), which suggests that glycolysis is not active in boar sperm when just ejaculated. Thus, during the dilution process, if the boar semen was diluted with a high-glucose medium, the boar sperm might suffer from high glycolysis stress, thus causing decreased sperm quality. The sperm membrane integrity and acrosome integrity in the high-glucose pretreatment in this study were lower than those in the treatment with 30.6 mM glucose (low glucose), thus agreeing with it. Therefore, a high-glucose medium could stimulate sperms with high glycolysis that would lead to disruption of sperm cellular homeostasis and decreased sperm quality and function.

On a worldwide basis, around 99% of artificial inseminations in pigs are done with liquid semen stored at 17°C, while <1% of frozen–thawed sperm are used (29). The problems associated with cryopreservation procedures and their post-thaw results limit the use of frozen–thawed sperm. The frozen–thawed sperm still show a lower survival rate, or the surviving sperm shows a shorter lifespan *in vitro* (36) compared to liquid-stored sperm. Thus, artificial insemination with frozen–thawed sperm has obviously resulted in a low farrowing rate (70–80%). Meanwhile, >90% is achieved by conventional AI of liquid-stored semen under controlled breeding management conditions (4, 8). In addition, the artificial insemination of frozen–thawed sperm has resulted in smaller litter sizes, usually 2 to 3 piglets fewer than that when liquid-stored sperm was used (8, 9), indicating that preventing boar sperm cryo-damage during the cryopreservation process is still a critical challenge. Although several studies have tested additives and changes in the speed of cooling and thawing to improve the boar sperm quality in recent years (6, 19), the overall procedure is basically still the same, which is just a simple modification of the protocol reported by Westendorf almost 40 years ago (37). Developing a novel and effective method for boar sperm cryopreservation might help to improve the post-thaw sperm quality, and it has become vital for the use of frozen–thawed sperm in pig artificial insemination. As we all know, keeping the sperm with good function before cryopreservation is generally a benefit for improving the post-thaw sperm. If the sperm was excessively activated before cryopreservation, the post-thaw sperm quality was decreased. In the present study, we found that the post-thaw sperm quality patterns, such as the total motility, progressive motility, VSL, membrane integrity, and acrosome integrity, were significantly increased by decreasing the glucose level in the pre-treatment medium as the low-glucose pre-treatment suppressed the sperm from high glycolysis activation, while the high-glucose medium induced sperm glycolysis. Therefore, during the cooling process, using low-glucose medium diluted with freshly ejaculated semen helps to protect boar sperm function and thus improve the post-thaw sperm quality.

Interestingly, when we analyzed the activities of SDH and MDH, which are two key enzymes of OXPHOS, it was found that those enzymes' activities were increased in the low-glucose treatment compared to that in the high-glucose treatment. It might be because the sperm mitochondria were protected from cryo-damage in the low-glucose treatment. The result of the mitochondrial membrane potential was consistent with it. Besides that, in this study, the low-glucose treatment also decreased the post-thaw sperm apoptosis. It agreed with a report that sperms with high activation led to a decreased sperm lifespan during liquid storage *in vitro* (5, 38). Based on those results, before cryopreservation, dilution of the ejaculated semen with a low-glucose medium is essential to improve the post-thaw sperm mitochondrial function.

**Figure 7:**
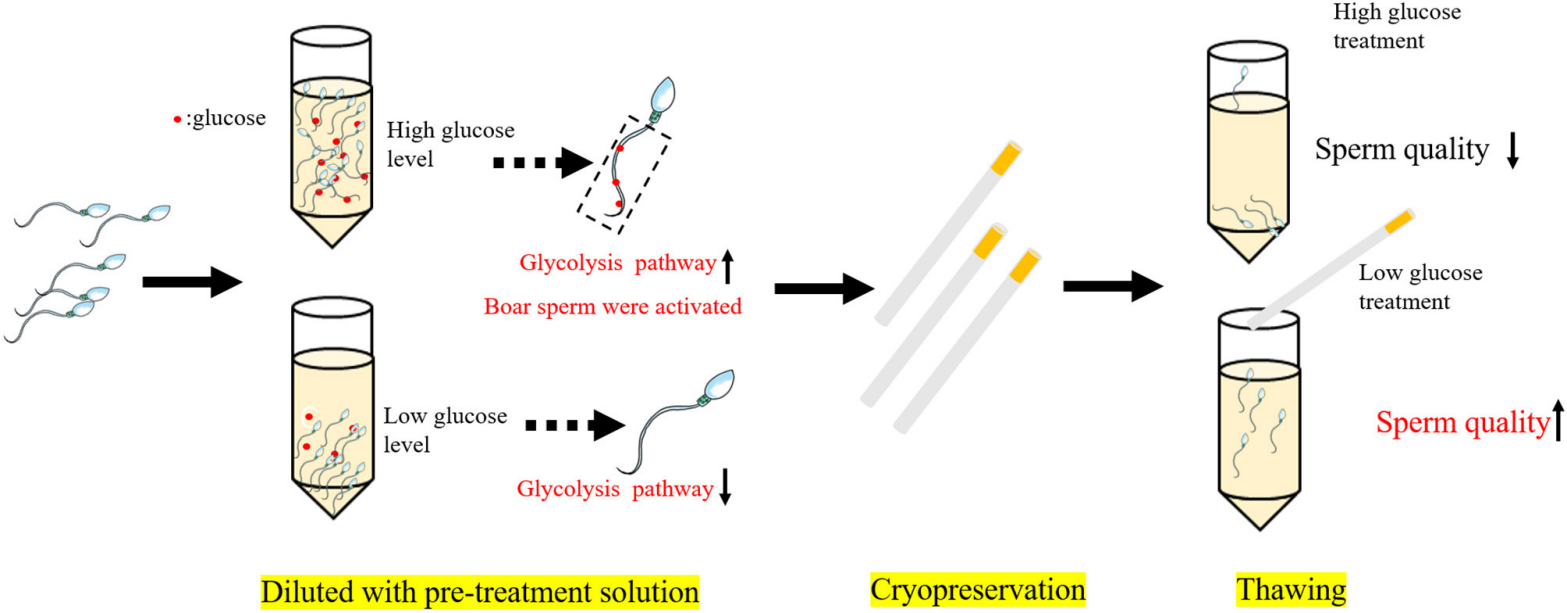
Mechanisms of reducing the glucose level in the pre-treatment solution improve the boar post-thaw sperm quality. High glucose level in the pretreatment solution enhances the stimulation of the glycolysis pathway and led to the activation of boar sperm before cryopreservation, thus resulting in decreased boar post-thaw sperm quality, whereas reduction of the glucose level would inactivate the glycolysis pathway to increase the post-thaw sperm quality during cryopreservation.

In conclusion, as shown in Figure 7, during the cooling process, a high glucose level in the pre-treatment solution enhances the stimulation of the glycolysis pathway and led to activation of the boar sperm before cryopreservation, thus result in decreased boar post-thaw sperm quality, whereas reducing the glucose level would inactivate the glycolysis pathway to increase the post-thaw sperm quality during cryopreservation.

## Data Availability Statement

The original contributions presented in the study are included in the article.

## Ethics Statement

The animal study was reviewed and approved by Qingdao Agriculture University Institutional Animal Care and Use Committee.

## Author Contributions

ZZ was responsible for experimental design, sample, data analysis, and writing of the manuscript. WZh and RL were responsible for sample collection and data analysis. WZe was responsible for the discussion about the experimental design. All authors contributed to the article and approved the submitted version.

## Funding

This work was supported in part by the Start-up Found for High-level Talents of Qingdao Agricultural University for ZZ (1121010), the Shandong Province Central Guided Local Science and Technology Development Project for ZZ (YDZX2021113), and the National Key R&D Program of China (number 2018YFD0501000) for WZe.

## Conflict of Interest

The authors declare that the research was conducted in the absence of any commercial or financial relationships that could be construed as a potential conflict of interest.

## Publisher's Note

All claims expressed in this article are solely those of the authors and do not necessarily represent those of their affiliated organizations, or those of the publisher, the editors and the reviewers. Any product that may be evaluated in this article, or claim that may be made by its manufacturer, is not guaranteed or endorsed by the publisher.
